# ESAMask: Real-Time Instance Segmentation Fused with Efficient Sparse Attention

**DOI:** 10.3390/s23146446

**Published:** 2023-07-16

**Authors:** Qian Zhang, Lu Chen, Mingwen Shao, Hong Liang, Jie Ren

**Affiliations:** College of Computer Science and Technology, China University of Petroleum (East China), Qingdao 266580, China; zhangqian8266@163.com (Q.Z.); smw278@126.com (M.S.); liangh@upc.edu.cn (H.L.); z20070069@s.upc.edu.cn (J.R.)

**Keywords:** instance segmentation, sparse attention, related semantic awareness, mixed receptive field, context awareness, feature aggregation

## Abstract

Instance segmentation is a challenging task in computer vision, as it requires distinguishing objects and predicting dense areas. Currently, segmentation models based on complex designs and large parameters have achieved remarkable accuracy. However, from a practical standpoint, achieving a balance between accuracy and speed is even more desirable. To address this need, this paper presents ESAMask, a real-time segmentation model fused with efficient sparse attention, which adheres to the principles of lightweight design and efficiency. In this work, we propose several key contributions. Firstly, we introduce a dynamic and sparse Related Semantic Perceived Attention mechanism (RSPA) for adaptive perception of different semantic information of various targets during feature extraction. RSPA uses the adjacency matrix to search for regions with high semantic correlation of the same target, which reduces computational cost. Additionally, we design the GSInvSAM structure to reduce redundant calculations of spliced features while enhancing interaction between channels when merging feature layers of different scales. Lastly, we introduce the Mixed Receptive Field Context Perception Module (MRFCPM) in the prototype branch to enable targets of different scales to capture the feature representation of the corresponding area during mask generation. MRFCPM fuses information from three branches of global content awareness, large kernel region awareness, and convolutional channel attention to explicitly model features at different scales. Through extensive experimental evaluation, ESAMask achieves a mask AP of 45.4 at a frame rate of 45.2 FPS on the COCO dataset, surpassing current instance segmentation methods in terms of the accuracy–speed trade-off, as demonstrated by our comprehensive experimental results. In addition, the high-quality segmentation results of our proposed method for objects of various classes and scales can be intuitively observed from the visualized segmentation outputs.

## 1. Introduction

Instance segmentation is a challenging task in computer vision that aims to make pixel-level dense predictions and distinguish different instances in images. Driven by the progress of the information age and the practical needs of various application scenarios, instance segmentation has gained wide-ranging application demands and promising prospects across diverse industrial and daily-life domains. Notably, in autonomous driving [1], instance segmentation plays a pivotal role in assisting driving systems to recognize distinct lane markings, vehicles, pedestrians, and obstacles, thus enabling an accurate assessment of the surrounding driving environment. Similarly, within industrial production settings, real-time and precise segmentation of objects captured in video frames from work sites can effectively mitigate safety risks and enhance production efficiency. Furthermore, in areas such as medical image segmentation [2] and image editing and enhancement, the quest for faster and more accurate segmentation results remains a constant aspiration. These compelling factors motivate our research and development efforts aimed at devising an instance segmentation method that optimally balances speed and accuracy. 

Recent advancements in deep convolutional networks have led to the development of two-stage models [3,4,5,6,7,8] such as Mask RCNN and single-stage methods [9,10,11,12,13] such as YOLACT for instance segmentation. The single-stage methods offer faster inference times [14] due to their end-to-end architecture, making them more suitable for practical scenarios. In recent years, the YOLO series of object detection models [15,16,17,18,19], renowned for their fast and accurate performance, have also developed variants adapted for segmentation tasks, which further propel the advancement of instance segmentation. However, there is still room for improvement in the segmentation accuracy of single-stage methods. This motivates us to think about a question: can we use the advantage of single-stage real-time and add new mechanisms to improve its segmentation accuracy? 

With the emergence of vision transformers [20] in the field of computer vision, several models based on vision transformers, such as Mask Transfiner [21], QueryInst [22], SOLQ [23], and Mask2Former [24], have achieved breakthroughs in segmentation accuracy. Self-attention, a core component of transformers [25], allows for better capturing of long-range dependencies compared to convolutions. However, using global self-attention throughout the feature extraction process increases the computational complexity and memory usage of the model exponentially with the input feature map resolution. This poses challenges for training the model on ordinary hardware devices and results in unsatisfactory inference times for downstream tasks. 

To address this problem, researching sparse attention strategies as alternatives to global attention has become a promising direction. In recent years, significant progress has been made in the development of sparse attention mechanisms. The pioneering work of Swin Transformer [20] introduced the use of local and shifted windows for self-attention computations, leading to a significant reduction in computational costs. NAT [26] extracts features by conducting dot product operations within a window defined by each pixel and its nearest neighbors. DiNAT [27] expands the receptive field by introducing dilation operations based on NAT. Despite employing diverse sparse techniques for key-value pair selection, all of the mentioned methods depend on manually defined rules to determine attention regions, resulting in the sharing of selected key-value pairs among query regions. This indiscriminate application of sparse attention in each sub-region fails to attend to different targets differentially. This inspired us to contemplate the second question: can a novel sparse attention mechanism be designed to enable the model to perceive different semantic regions and adaptively search for attention windows? 

Furthermore, we noted that the aforementioned models conduct attention operations using a fixed window size, which imposes constraints on capturing features for objects of different sizes. Hence, this motivates us to explore how to simultaneously model global, regional, and local information to better adapt to mask prediction for objects of different sizes.

To tackle these challenges and questions, this paper proposes a real-time segmentation model called ESAMask. The objective is to improve the accuracy of the model while ensuring real-time performance. Combining thoughts on problem one, the proposed model follows the design paradigm of a single-stage model and introduces novel modules that are efficient and memory-friendly. 

To address the second question, the paper introduces the Related Semantic Perceived Attention module (RSPA), which dynamically adapts to different semantic regions. RSPA performs coarse-grained correlation calculations in sub-regions of the graph to reserve a few key-value regions with high semantic correlation in each query region. Fine-grained attention operations are then performed on these relevant regions, strengthening the semantic representation of feature maps. 

For the third question, considering that targets in the image have different sizes, the paper designs the Mixed Receptive Field Context Perception Module (MRFCPM). This module fuses information from three branches: global content awareness, large-kernel region awareness, and convolutional channel attention. By explicitly modeling information in global, regional, and local scales, this module improves the segmentation accuracy of multi-scale objects.

In addition, to further reduce the weight of the model, the paper introduces GSInvSAM in the network neck part. GSInvSAM reduces redundant information and enhances channel information interaction by utilizing GSConv [28] and inverted bottleneck structures. Leveraging SAM’s [29] non-parametric attention, it assists the pyramid network in focusing on key feature areas without increasing computational costs. 

Combining the above analysis and strategies, the contributions of this paper are summarized as follows:

(1) We introduce RSPA to the backbone network, which supports differentiated attention for different semantic features in a sparse, adaptive manner.
(2) We design GSInvSAM, which removes redundant information and strengthens feature associations between different channels during bidirectional pyramid feature aggregation.
(3) We added the MRFCPM to the prototype branch, which performs multi-level modeling of global, regional, and local representations, which helps to improve the segmentation effect of targets of different scales.
(4) The design of the entire model and each component follows the principles of being lightweight, effective, and efficient. Experimental results show that our model achieves a better balance between accuracy and efficiency.

## 2. Related Work

### 2.1. Instance Segmentation

Instance segmentation, similar to the object detection task, can be categorized into two-stage and single-stage methods. The two-stage instance segmentation first extracts a region of interest (ROI) based on the features learned by the network and then segments each instance on the ROI [30]. Mask RCNN [3] is the most classic two-stage instance segmentation model, which adds FCN [31] branches to predict masks based on Faster RCNN [30]. The remarkable segmentation performance of Mask RCNN has spurred a wave of technological advancements in the field of instance segmentation. Subsequently, various extension methods based on Mask RCNN [3] have emerged. PANet [4] introduces a bottom-up path to FPN and integrates low-level, high-resolution detailed feature information into the high-level semantic feature map, thus enhancing the fine-grained segmentation of instances. BMask [8], BPR [7], RefineMask [32], and SharpContour [33] refine the segmentation mask of Mask RCNN by focusing on boundary refinement. MS RCNN [5] argues that classification confidence does not directly correlate with segmentation accuracy. To address this, a new MaskIOU branch is introduced, combining the prediction score and classification score to evaluate the effectiveness of mask generation. These models contribute to the enrichment of the two-stage Mask RCNN family from various perspectives. However, two-stage segmentation methods rely on the selection of a large number of regions of interest, which to some extent affects the inference speed of the model and fails to meet the speed requirements in practical applications.

Due to the slow inference speed of the two-stage method and the insufficient simplicity of the model, the single-stage end-to-end segmentation model has gradually attracted researchers’ interest. YOLACT [9] stands as the pioneering single-stage instance segmentation model that achieves true real-time performance. Its real-time segmentation capability is enabled through a simple design that combines the mask coefficient branch and the prototype branch to generate masks. The success of YOLACT has inspired researchers to focus on enhancing the network’s reasoning speed while simultaneously improving the accuracy of model segmentation. BlendMask [13] follows the design idea of YOLACT [9], removes redundant mask branches, and designs a reasonable blender module to fuse high-level attention branches and low-level details. CenterMask [34] achieves simple, effective, and real-time segmentation by incorporating a spatial attention-guided masking branch into the anchor-free detector. By considering that different instances occupy distinct locations, Wang et al. propose the segmentation of instances based on location prediction. Hence, the devised SOLO [35] network partitions the input image into grids of size S × S, wherein each grid classifies and segments the contained objects. SOLOv2 [36] adds a dynamic convolution kernel to predict the parameters of the mask head on the basis of SOLO, which further improves the effect of instance segmentation. The aforementioned single-stage methods develop compact and end-to-end network architectures from various angles, continually enhancing their inference speed, which is beneficial for real-time operation of models in practical application scenarios or on mobile devices. Nevertheless, a good model should prioritize both fast execution speed and improved segmentation accuracy. Therefore, this paper leverages the real-time advantages of single-stage models to design an end-to-end network architecture and introduces novel modules to enhance its performance in terms of accuracy. 

In recent years, the success of self-attention in computer vision has sparked increased interest in self-attention-based instance segmentation methods. SOLQ [23], proposed by Dong et al., employs a self-attention-based Swin Transformer [20] for feature extraction. The extracted features are then fed into a unified query head, enabling joint prediction of categories, locations, and instances. QueryInst [22], designed by Fang et al., builds upon Sparse R-CNN [37] and incorporates query embedding and dynamic convolution modules for multi-task learning. Mask2Former [24] introduces a masked attention approach to replace a portion of self-attention for decoding multi-level features and directly predicting instance and semantic masks. Compared to CNN-based models, the aforementioned self-attention-based segmentation models capture features at a global scale, resulting in higher segmentation accuracy. However, the self-attention mechanism computes affinities among all features, leading to significant memory consumption and computational costs, thereby increasing the training difficulty. Therefore, this paper focuses on lightweight and sparse attention methods and proposes and introduces an efficient and dynamic sparse attention mechanism to maximize the benefits of attention operations on model performance while ensuring memory-friendly and real-time inference. 

### 2.2. Attention for Instance Segmentation

The remarkable feature extraction capabilities of self-attention have made its variants immensely popular in various vision domains. Liu et al. introduced the Swin Transformer [20], which efficiently reduces the computational burden of global attention by incorporating self-attention and pixel-shifting self-attention operations on non-overlapping sub-windows. Consequently, it has become a widely adopted backbone network for diverse visual tasks. Notably, SOLQ [23], Mask2Former [24], and Mask DINO [38] are exemplary instance segmentation algorithms that leverage the Swin Transformer [20] to extract features and achieve competitive segmentation outcomes. 

The idea of window-based self-attention inspired some later work. NA [26] adopts a sliding window approach to perform self-attention within a window comprising each pixel and its neighboring pixels. This allows each pixel to modify its receptive field without pixel shifting while maintaining translation invariance. However, window attention fails to capture long-range interdependencies, leading to the proposal of DiNA [27]. DiNA presents a flexible and efficient extension of NA by increasing the step size, akin to dilated convolution, to expand the window attention range and receptive field without incurring additional computational costs. These methods share a common characteristic: attention is applied uniformly across the entire feature map in window units. Nonetheless, this attention approach treats each window equally, disregarding the discriminative impact of semantics on different targets. Considering this limitation, this study delves into a dynamic and adaptive semantic-relevant sparse attention method. This method enables different query windows to focus on semantically relevant regions with higher correlation, thereby enhancing the utilization of semantic information for diverse targets. 

In response to the requirements of target discrimination and dense prediction in instance segmentation, Nguyen et al. introduced BoxeR [39]. BoxeR is a method that generates interest boxes by employing box attention within a predefined reference window and predicting its geometric transformation. By enabling spatial interaction between grid features and attention operations from these interest boxes, BoxeR proves advantageous for end-to-end object detection and instance segmentation tasks. However, the dependence on a predetermined reference window size limits its adaptability to objects of varying sizes, posing algorithmic constraints. Instead, we propose a mixed receptive field module that combines sparse global attention, window attention, and channel attention, facilitating the capture of feature information of corresponding sizes for objects of different scales. 

## 3. Methods

### 3.1. Overall Architecture

**Figure 1:**
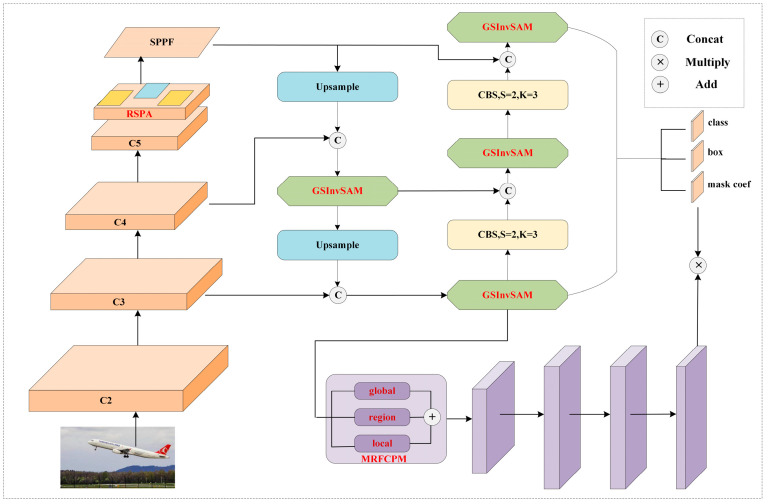
The overall architecture of ESAMask. The red bolded parts represent the modules proposed in this paper.

To leverage the simplicity and fast inference speed of single-stage segmentation models while incorporating the advantages of self-attention and long-range modeling, this paper introduces an effective and efficient real-time segmentation network called ESAMask. The network architecture is depicted in Figure 1. The backbone network encodes the input image across multiple stages, gradually transforming spatial information into high-dimensional channel information. By integrating the designed RSPA module into the feature map downsampled by a factor of 32, the network can effectively capture semantic variations during feature extraction without introducing excessive parameters. To enhance feature fusion across different scales, this study adopts a conventional two-way pyramid structure. However, a novel GSInvSAM is proposed in this work to replace the commonly used CSP block. This novel module facilitates effective information fusion and interaction among different feature layers while reducing redundant parameters and computational costs. In the prediction head section, an anchor-free decoupling head is employed to perform classification and detection tasks, reducing the post-processing time associated with non-maximum suppression (NMS). For the segmentation task, the prototype branch is primarily responsible for mask prediction. Given the significance of fully utilizing features in generating accurate masks, a lightweight MRFCPM is designed and integrated into the prototype branch to cater to the diverse range of feature representations required for targets of different scales.

### 3.2. Related Semantic Perceived Attention

**Figure 2:**
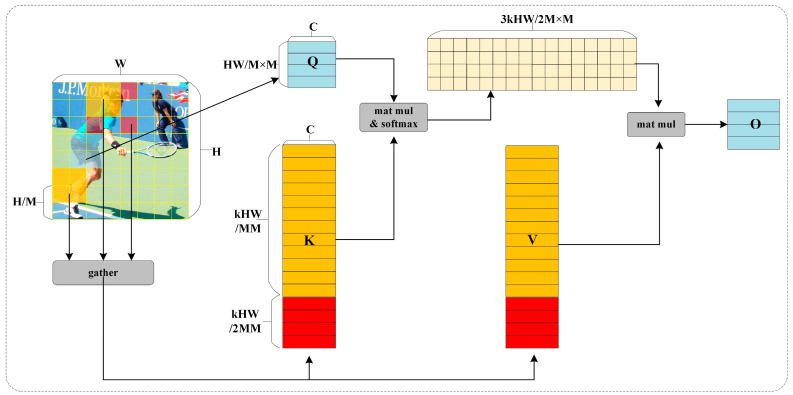
Illustration of the execution process of Related Semantic Perceived Attention. Blue represents the query area; yellow represents the first k semantically related areas; red represents the expansion area corresponding to the last k/2 related regions; and the yellow and red regions are aggregated into the key-value region corresponding to the query region. RSPA enables GPU-friendly sparse attention operations by aggregating semantically related regions of the same target.

Several current works have designed different windowed attention or sparse attention mechanisms to alleviate the computationally intensive problem of MHSA. However, most of them are based on artificially set fixed rules that share a subset of key-value pairs within all regions indiscriminately and cannot perceive the semantic relevance of targets in different regions. In this work, we explore a dynamic adaptive and semantically relevant sparse attention mechanism to design the RSPA module. The main idea of RSPA is to initially find the top k + k/2 semantically relevant sub-regions corresponding to each region within all sub-regions globally, remove the irrelevant or less relevant regions, and finally perform token attention operations within the semantically relevant regions retained in each region. The execution process of RSPA is shown in Figure 2.

**Region division and related region search.** For the input feature map X, we divide it into M × M non-overlapping grids. By linearly mapping the partitioned X, the Query, Key, and Value tensors are obtained ($Q,K,V\in R^{M^{2}\times \frac{HW}{M^{2}}\times C}$). In order to establish semantic associations for each region, this paper uses a directed graph to construct an adjacency matrix. Specifically, firstly, the average value of each region is calculated to obtain the region-level $Q^{m},K^{m}\in R^{M^{2}\times C}$. Then, the affinities between different regions are obtained by matrix multiplication to construct an adjacency matrix $A^{m}\in R^{M^{2}\times M^{2}}$. This process can be represented as follows:

(1) $$A^{m}=Q^{m}{\left(K^{m}\right)}^{T}$$

where $A^{m}$ represents the semantic correlation between the two regions and T represents the matrix transpose.

Next, we crop the adjacent region and perform row-level top-k operations to obtain a semantically related index matrix $S^{m}\in R^{M^{2}\times k}$. The formula is as follows:

(2) $$S^{m}=IndexofTopk\left(A^{m}\right)$$

where the IndexofTopk operation retrieves the indices of the top k regions with the highest relevance to each query region, based on the magnitudes of the affinity matrix $A^{m}$.

Among the k correlation regions, the regions with higher correlation values are most likely to be located inside the same target, and the regions with the next highest correlation values, such as the *k*th and *k* − 1th regions, are likely to be located near the target boundary. In order to improve the perception of the contextual information inside and outside the target boundary during network feature extraction, we borrow the idea of expansion convolution and add the expansion regions $D^{m}\in R^{M^{2}\times k/2}$ corresponding to the latter *k*/2 relevant regions to the semantic relevant regions, where *k*/2 is rounded down when k is odd.

**Associated region token attention.** According to the index matrix $S^{m}$ and the corresponding expansion region $D^{m}$, we can perform token-level attention operations on the joint key-value pairs of the *i*th query region and its corresponding top *k* + *k*/2 semantically related regions $S_{\left(i,1\right)}^{m},\cdots ,S_{\left(i,k\right)}^{m},D_{\left(i,1\right)}^{m},\cdots ,D_{\left(i,k/2\right)}^{m}$. Since the relevant regions are scattered in different parts of the entire feature map, it will be very inefficient if the query region is followed by the key-value region for attention operation. Therefore, before the attention operation, we first aggregate key-value pair tensors of relevant regions to perform GPU-friendly token attention.

The process of the aggregation operation is shown in Formulas (3) and (4):

(3) $$K^{g}=gather\left(K,S^{m}+D^{m}\right)$$

(4) $$V^{g}=gather\left(V,S^{m}+D^{m}\right)$$

where the gather operation represents the aggregation of the scattered related regions $S^{m}$ and $D^{m}$ corresponding to the same query region, $K^{g}\in R^{M^{2}\times \frac{\left(k+k/2\right)HW}{M^{2}}\times C}$ is the key tensor after aggregation, and $V^{g}\in R^{M^{2}\times \frac{\left(k+k/2\right)HW}{M^{2}}\times C}$ is the value tensor after aggregation.

The process of token attention can be expressed as Equation (5):

(5) $$O=softmax\left(\frac{Q{\left(K^{g}\right)}^{T}}{\sqrt{C}}\right)\;V^{g}$$

where C represents the number of channels, which is used to avoid gradient disappearance and concentration of weights.

### 3.3. GSInvSAM

**Figure 3:**
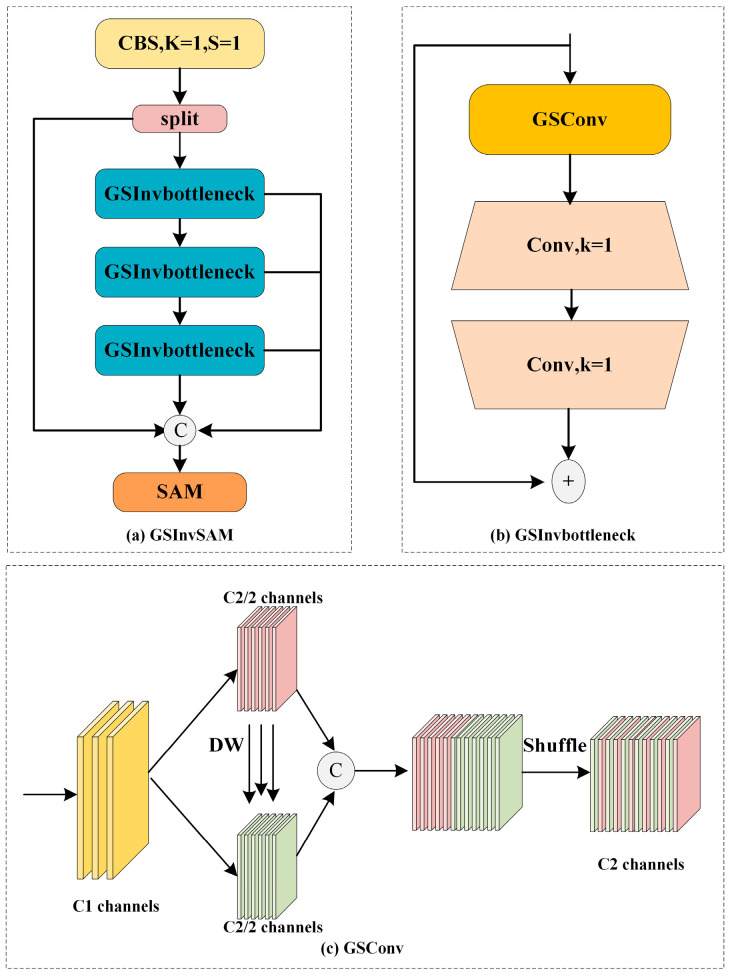
The structure of the (**a**) GSInvSAM, (**b**) GSInvbottleneck, and (**c**) GSConv.

The backbone network is usually used as an encoder to extract image features. As the model level deepens, spatial information is gradually converted to channel information, and the nonlinear expression ability of the model is becoming stronger and stronger. To fuse backbone feature information at different scales, various feature pyramid networks are widely used. However, directly splicing the feature maps of two adjacent layers will inevitably bring about the problems of information redundancy and lack of interaction between channels. In order to alleviate the above problems by processing the feature maps of neck stitching, we propose the GSInvSAM structure based on GSConv [28], inverted bottleneck, and SimAM [29], as shown in Figure 3.

GSInvBottleneck is the basic block of GSInvSAM. It consists of a GSConv [28] and two symmetric kernel-1 convolution operations. Among them, GSConv compresses redundant information by halving the number of channels and deep-wise operations and performs shuffle operations on channel features to enhance feature interaction. After GSConv, a symmetric convolution operation of 1 × 1 channel expansion and channel compression is performed to further strengthen the fusion of channel information. Borrowing ideas from OSA [40], we aggregate multiple depths of GSInvBottleneck to generate richer gradient flow information. In addition, we added the Simple Attention Module [29] at the end of GSInvSAM. Based on the principle of the optimal solution of the energy function, SimAM [29] assigns different weights to each pixel value of the feature map, which can capture important feature representations without increasing any parameters.

### 3.4. Global Content-Aware Module

**Figure 4:**
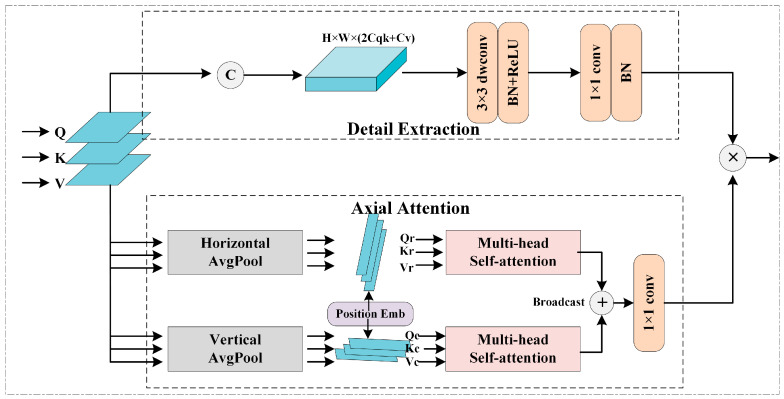
The calculation process of the Global Content-aware Module.

Self-attentive mechanisms have achieved remarkable success in capturing long-range dependencies, especially for intensive prediction tasks. However, due to its large number of model parameters, it inevitably leads to an exponential increase in computational cost and memory usage. In order to model global information while improving the inference efficiency of the model, this paper proposes a memory-friendly Global Content-aware Module, which contains a lightweight and efficient axial attention branch for extracting global semantics and a detail extraction branch based on small kernel convolution to retain local details. The structure of GCAM is shown in Figure 4.

**Axial Attention.** To extract global contextual information with low computational cost, we perform self-attention operations on the horizontal and vertical axes separately and aggregate information from both directions. Specifically, we convert the input feature map X into a Query, Key, and Value tensor. In the direction of the horizontal axis, we perform average pooling on each row of feature tensors to obtain $Q_{\left(r\right)},K_{\left(r\right)},V_{\left(r\right)}\in R^{H\times C_{qk}}$. The calculation process of $Q_{\left(r\right)},\;\;K_{\left(r\right)}$, and $V_{\left(r\right)}$ can be expressed as follows:

(6) $$Q_{\left(r\right)}={\left(\frac{1}{W}\overset{W}{\underset{j=1}{\sum }}Q\left(1,j\right),\cdots ,\frac{1}{W}\overset{W}{\underset{j=1}{\sum }}Q\left(r,j\right)\right)}^{T}$$

(7) $$K_{\left(r\right)}={\left(\frac{1}{W}\overset{W}{\underset{j=1}{\sum }}K\left(1,j\right),\cdots ,\frac{1}{W}\overset{W}{\underset{j=1}{\sum }}K\left(r,j\right)\right)}^{T}$$

(8) $$V_{\left(r\right)}={\left(\frac{1}{W}\overset{W}{\underset{j=1}{\sum }}V\left(1,j\right),\cdots ,\frac{1}{W}\overset{W}{\underset{j=1}{\sum }}V\left(r,j\right)\right)}^{T}$$

where W denotes the width of the image, j denotes the jth column of the image, and r denotes the total number of rows of the image.

In the direction of the vertical axis, we perform the same operation on the elements of each column to obtain $Q_{\left(c\right)},K_{\left(c\right)},V_{\left(c\right)}\in R^{W\times C_{qk}}$. The calculation process of $Q_{\left(c\right)},\;\;K_{\left(c\right)}$, and $V_{\left(c\right)}$ can be expressed as follows:

(9) $$Q_{\left(c\right)}=\left(\frac{1}{H}\overset{H}{\underset{i=1}{\sum }}Q\left(i,1\right),\cdots ,\frac{1}{H}\overset{H}{\underset{i=1}{\sum }}Q\left(i,c\right)\right)$$

(10) $$K_{\left(c\right)}=\left(\frac{1}{H}\overset{H}{\underset{i=1}{\sum }}K\left(i,1\right),\cdots ,\frac{1}{H}\overset{H}{\underset{i=1}{\sum }}K\left(i,c\right)\right)$$

(11) $$V_{\left(c\right)}=\left(\frac{1}{H}\overset{H}{\underset{i=1}{\sum }}V\left(i,1\right),\cdots ,\frac{1}{H}\overset{H}{\underset{i=1}{\sum }}V\left(i,c\right)\right)$$

where H denotes the height of the image,  i denotes the *i*-th row of the image, and c denotes the total number of columns of the image.

To make feature tensors position sensitive, we introduce axis position embeddings to sense the position of features. The position embedding vector $E_{\left(r\right)}^{q},E_{\left(r\right)}^{k}\in R^{H\times C_{qk}}$ is constructed by randomly initializing learnable parameters $N_{\left(r\right)}^{q},N_{\left(r\right)}^{k}\in R^{L\times C_{qk}}$ and performing linear interpolation. In the same way, $E_{\left(c\right)}^{q},E_{\left(c\right)}^{k}\in R^{W\times C_{qk}}$ can be obtained. During the model training process, the position vector can be dynamically updated according to the actual features. Position-aware axis attention can be expressed as the formula (12):

(12) $$\begin{array}{cc} y\left(i,j\right)= & \overset{H}{\underset{p=1}{\sum }}softmax_{p}\left({\left(Q_{\left(r\right)i}+E_{\left(r\right)i}^{q}\right)}^{T}\left(K_{\left(r\right)p}+E_{\left(r\right)p}^{k}\right)\right)V_{\left(r\right)p}\\ & +\overset{W}{\underset{p=1}{\sum }}softmax_{p}\left({\left(Q_{\left(c\right)j}+E_{\left(c\right)j}^{q}\right)}^{T}\left(K_{\left(c\right)p}+E_{\left(c\right)p}^{k}\right)\right)V_{\left(c\right)p} \end{array}$$

where p represents the position of the pixel, i represents the horizontal coordinate of the pixel point, j represents the vertical coordinate of the pixel point, and E represents the position vector, which is added to the query tensor *Q* and key tensor *K* to sense the position information of the feature map.

The horizontal and vertical tensors with embedded location information are fed separately into the multi-headed attention module for self-attentive operations. To combine the feature information in both directions to model the global information, we fuse the horizontal and vertical features using a simple and efficient broadcast operation. The time complexity of the axial average pooling is $O\left(H+W\right)\left(2C_{qk}+C_{v}\right)$, and the time complexity of the self-attention is $O\left(H^{2}+W^{2}\right)\left(C_{qk}+C_{v}\right)$. Thus, the axial attention branching significantly reduces the time complexity of modeling global dependencies.

**Detail Extraction.** To compensate for the local details lost when global extraction is performed by axis attention, we designed the Detail Extraction branch to capture and preserve local information. As shown in Figure 4, the *Q*, *K*, *V* tensor is stitched in the channel dimension, and local features are extracted by a small kernel depth separable convolution of 3 × 3. Then, the point convolution with kernel 1 and the corresponding normalization and activation operations are used to reduce the channel dimension to C. Finally, the Detail Extraction branch and Axial Attention branch are fused in a multiplicative manner to achieve a mutual complement of global and local information.

### 3.5. Mixed Receptive Field Context Perception Module

**Figure 5:**
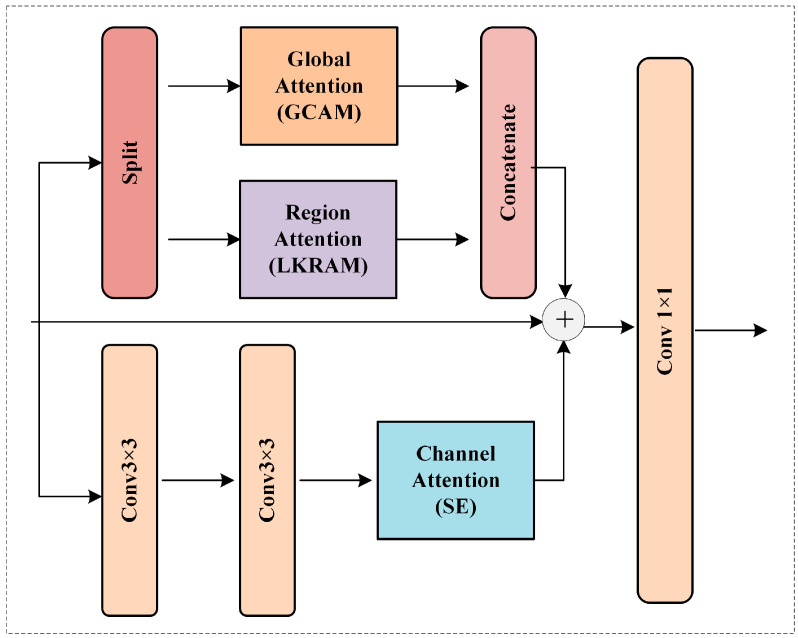
The pipeline of the Mixed Receptive Field Context Perception Module.

The generation of prototypes plays a key role in the quality of instance segmentation, and different prototypes represent different instance information in feature maps. In order to make the prototype branch of the head be able to fully extract and preserve the features of the backbone encoding, a novel Mixed Receptive Field Context Perception Module is designed in this paper. It can jointly capture global, regional, and local representation information, which is helpful for the segmentation of objects at different scales. The structure of MRFCPM is shown in Figure 5.

**Figure 6:**
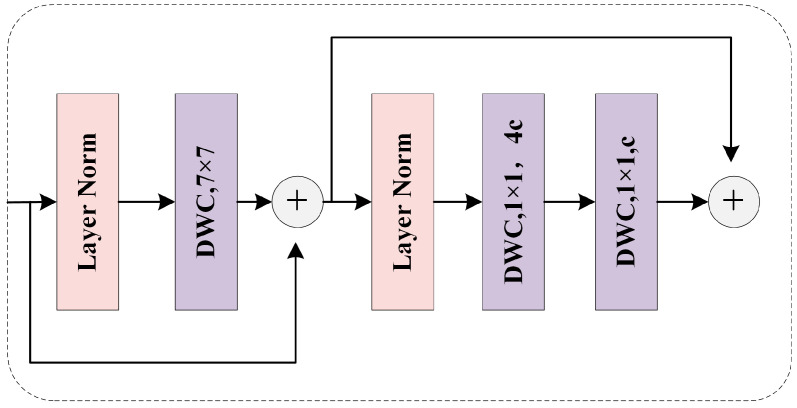
The structure of Large Kernel Region-aware Module.

The whole module mainly includes three branches of global attention, regional attention, and channel attention to extract key representation information of large-scale, medium-scale, and small-scale ranges, respectively. The global attention part uses the lightweight GCAM designed in this paper to model large-scale and long-distance information dependencies. For small-scale targets or local details, standard convolution can play a good role in feature extraction. Therefore, we directly use ordinary convolution with a kernel of 3 to capture local features and use a simple SE channel attention block to strengthen the interaction of channel information in key dimensions. For the extraction of regional features, the most commonly used is Window Attention. However, in order to reduce computational costs and maintain the overall lightweight and efficient nature of the model, this paper did not adopt the approach of window attention. Instead, a Large Kernel Region-aware Module was designed to extract crucial region-specific information. The structure of LKRAM is shown in Figure 6.

**Large Kernel Region-aware Module.** The larger receptive field is the reason why window attention has an advantage over ordinary convolution. However, the operation of self-attention in the whole window inevitably introduces a large amount of calculation. Inspired by large kernel convolution and depth convolution, this paper argues that large kernel depth convolution can provide a larger receptive field similar to window self-attention, while greatly reducing computational costs. Therefore, we use a large kernel (e.g., 7 × 7)-based depthwise convolution to extract region information. In addition, we use a depthwise convolution scaling with a kernel of 1 to perform dilation and compression operations on each channel to minimize the information redundancy between channels. In the whole module, two consecutive residual connections are used to ensure the stability of the gradient, and the Batch Norm (BN) commonly used in convolution is replaced with Layer Norm (LN) to avoid the problem of weak model generalization caused by BN.

## 4. Experiments

### 4.1. Dataset and Evaluation Metrics

The main experiments in this paper are conducted on the MS COCO2017 [41] dataset. MS COCO2017 contains 80 kinds of objects, including rich and colorful image data of different scenes in the real world, and is the most general and powerful benchmark dataset in instance segmentation tasks. The model is trained on a training set (train2017) containing 118 k images and validated on a validation set (val2017) containing 5 k images. The final results are evaluated on COCO val2017. All experiments are evaluated using COCO’s standard evaluation metrics, including mean average precision (mAP), AP_S_, AP_M_, and AP_L_. AP_S_ stands for small objects with a size smaller than 32 × 32; AP_M_ stands for medium objects with a size between 32 × 32 and 96 × 96; and AP_L_ stands for large objects with a size greater than 96 × 96. The model evaluates the inference speed using FPS (frames per second) and time (the time taken for processing a single image). In order to demonstrate the lightweight nature of the designed components in this study, we employ the metrics of Params (parameters) and GFLOPs to quantify the model’s parameter count and computational load, respectively.

### 4.2. Implementation Details

All experiments in this paper are conducted on a single NVIDIA 3090 GPU with a memory capacity of 24 GB. The ESAMask is implemented on the PyTorch 2.0 platform with CUDA 11.7. The training process consists of 200 epochs. During model training, the image size is set to 640; the batch size is set to 12; and the SGD optimizer is used for optimization. The initial learning rate is set to 0.01; momentum is set to 0.937; and weight decay is set to 0.0005. Various data augmentation strategies, such as photometric distortion, random flipping, and mosaic, are employed to enhance the robustness of the learned features. Specifically, photometric distortion transforms the input images into the HSV color space and modifies the values of the three channels (h, s, and v) by ratios of 0.015, 0.7, and 0.4, respectively. Horizontal flipping and mosaic operations are applied to the input images with probabilities of 0.5 and 1.0, respectively, to augment the dataset. Training the model on the COCO dataset takes approximately 7 to 9 days. Some parameters involved regarding the module proposed in this paper can have different effects on the experimental results. Therefore, in Section 4.4, we conduct a series of ablation experiments with different parameter settings to identify the optimal combination. Specifically, after comparing the results of multiple experiments, the model finally sets the window number M = 8 and k = 4 for RSPA; the expansion rate r of the reverse bottleneck for GSInvSAM is 2; and the convolution kernel size of LKRAM is 7 × 7.

### 4.3. Main Results

In this section, we compare ESAMask with other state-of-the-art methods on the COCO val set. 

**Figure 7:**
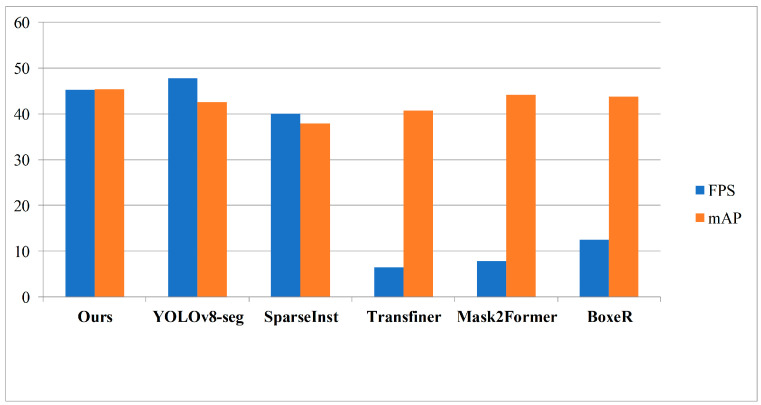
Speed–performance trade-off for various instance segmentation methods on COCO.

Figure 7 presents a comparison between our method and recent state-of-the-art segmentation models in terms of speed and performance. The statistical results depicted in the chart clearly demonstrate significant advantages of our model in terms of the trade-off between accuracy and efficiency, surpassing most advanced methods. More detailed quantitative comparison results are provided in Table 1 and Table 2.

The table data provide clear evidence of the competitive advantages of our model when compared to various state-of-the-art segmentation methods employing different design paradigms. Specifically, our proposed approach surpasses both two-stage methods, such as Mask RCNN [3], known for their high accuracy, and Transformer [25]-based methods such as Mask2Former [24], by achieving further improvements in accuracy while significantly outperforming them in terms of speed. These results underscore the robust feature representation capabilities of our model. Additionally, in contrast to renowned single-stage methods prioritizing speed, such as YOLACT [9], ESAMask maintains superior accuracy while exhibiting an approximate 10 FPS higher speed. This highlights the lightweight nature of the modules devised in this paper. By adhering to the principles of simplicity, effectiveness, and efficiency, our model outperforms the most recent sparse attention backbone network-based methods, including BoxeR [39], NA [26], and DiNAT [27]. Notably, our model demonstrates respective improvements in average precision (AP) of 1.6%, 0.9%, and 0.3% for each method and achieves a higher number of images detected per second compared to the aforementioned methods. Thus, our approach excels in both speed and accuracy.

Furthermore, the YOLO family has recently introduced the YOLO-seg [15,16] series specifically designed for real-time instance segmentation tasks, which has yielded remarkable outcomes. Comparing our method to YOLOv5-seg [16] and YOLOv8-seg [15], we have achieved respective improvements of 5.3% and 2.8% in mask mAP, albeit with a corresponding decrease in speed of approximately 2.5 FPS. This reduction in speed can be attributed to the additional memory access required by the introduced sparse attention mechanism, which marginally affects the inference speed. Nevertheless, our model remains capable of ensuring real-time operation while preserving its overall segmentation performance. We consider this minor decrease in speed to be reasonable. Additionally, by examining the indicators of AP_S_, AP_M_, and AP_L_ in Table 1, our method exhibits varying degrees of enhancement compared to the baseline model of YOLOv8-seg. This observation indicates that ESAMask is capable of delivering satisfactory detection results for objects of diverse sizes.

To demonstrate the lightweight nature of our proposed method more intuitively, this paper presents a statistical comparison of parameter count (Params) and floating-point operations (GFLOPs) among different state-of-the-art (SOTA) models. The results presented in Table 2 clearly indicate that our method achieves significant reductions in both Params and GFLOPs when compared to the latest window-sparse attention models, NA [26] and DiNA [27]. Furthermore, when compared to the baseline model, yolov8-seg [15], our method demonstrates similar Params and GFLOPs, thus indicating that the approach developed in this study aligns effectively with the intended objectives of being lightweight and efficient.

### 4.4. Ablation Study

**Effect of single modules.** To assess the efficacy of each proposed component in this study, we employ yolov8-seg [15] as the baseline model and integrate three distinct modules: RSPA, GSInvSAM, and MRFCPM. Table 3 illustrates the impact of incorporating RSPA, which enhances the model’s capacity to capture semantic information during feature extraction, resulting in a performance increase from 42.6 AP to 43.8 AP. Introducing the GSInvSAM structure in the neck region led to a reduction of 5.75 M parameters, a decrease of 19.3 GFLOPs, and an improvement of 0.7% in AP. This outcome highlights the simultaneous enhancement of performance and the elimination of redundant computations. Furthermore, by effectively modeling contextual information from various ranges, MRFCPM achieves a significant accuracy improvement of 0.9 points.

**Effect of combination modules.** To validate the synergistic effects of the individual components, we conducted a series of ablation experiments by combining the proposed modules. As shown in Table 3, when integrating RSPA and GSInvSAM into the baseline model, the mAP value increased by 2%, accompanied by a 0.5 FPS improvement. With the inclusion of all three modules, the model achieved a speed of 45.2 FPS while attaining a 45.3 AP. These ablation experiments demonstrate the effectiveness and efficiency of the designed modules in this study.

**Effect of M and k in RSPA.** RSPA requires querying k regions with high relevance from an M × M window to perform sparse attention. To investigate the influence of different values of M and k on model performance, we integrate the RSPA module into the yolov8-seg baseline model with varying M and k configurations. Table 4 presents the results of our experiments, indicating minimal variations in the model’s mAP across different combinations of M and k. Taking into account speed considerations, we achieve the highest value of 46.9 FPS when setting M to 8 and k to 4.

**Effect of GSInvSAM.** To explore effective methods for lightweight network neck design, we adopt yolov8-seg [15] as the baseline model and conduct a series of experiments by replacing its C2f module in the neck region with the GSInvSAM composed of different structures. As shown in Table 5, the model has the lowest number of parameters when Bottleneck consists of two consecutive GSConv [28], but there is a 0.5% AP degradation in model performance due to the loss of some valid information. When we combine GSConv with InvertConv, the model guarantees the inference performance while cutting down the redundant parameters. We also tried to increase the r of the inverted bottleneck from 2 to 4, and the number of model parameters increased by 3 M, but the performance did not improve significantly. Therefore, our model is set to r = 2. In addition, when we add the simple attention module [31] at the end of GSInvSAM, the model improves the performance by 0.5% without any increase in the number of parameters, which is very beneficial to the model.

**Effect of LKRAM kernel size.** LKRAM is a submodule in MRFCPM used to capture region representations. In order to set an appropriate convolution kernel size, we use the standard yolov8-seg model as the baseline and add MRFCPM with different k values to conduct multiple experiments. It can be seen from the experimental results in Table 6 that as the k value increases from 5 to 7, the mAP gradually increases. And at the same time, the speed decreases due to the increase in the number of parameters. When k increases from 7 to 9, the FPS decreases by 0.6, and the AP value only increases by 0.1%. Therefore, in order to ensure that the accuracy and speed can reach a more balanced state, the model finally sets k = 7.

### 4.5. Visualization of Results

**Figure 8:**
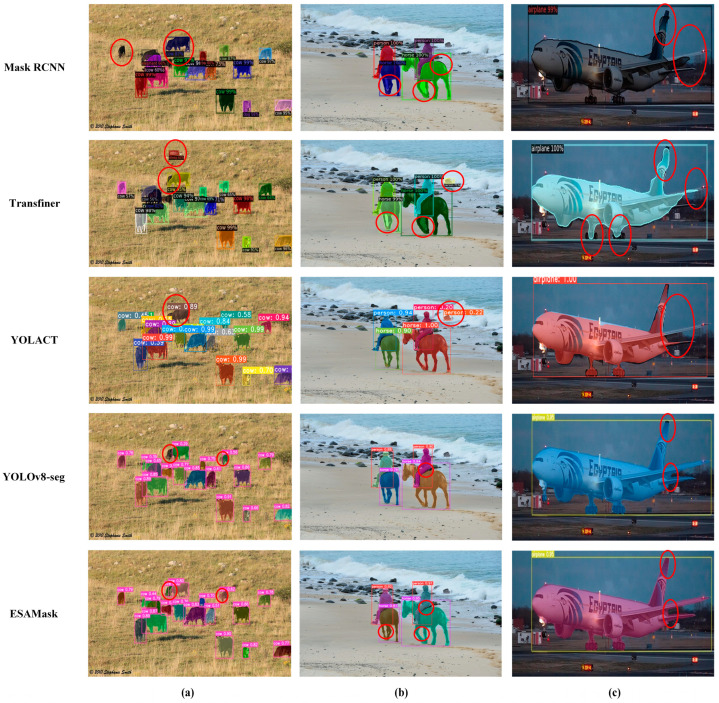
A visual comparison of ESAMask and other classical networks. (**a**) small targets; (**b**) medium targets; (**c**) large targets. The red circle represents a comparison of the detail part of the segmentation effect of different networks.

To provide a more intuitive demonstration of the mask generation quality of the proposed model, a qualitative comparison is conducted between ESAMask and other classical instance segmentation models. The results are presented in Figure 8, where the images are divided into three columns representing small, medium, and large targets, respectively. The visualization results clearly showcase that ESAMask outperforms other classical methods in terms of segmentation accuracy and quality across targets of varying scales. Notably, the model in this paper exhibits smoother and more detailed segmentation along the boundary pixels compared to Mask RCNN [3], Transfiner [21], and similar approaches. This can be observed in the horse leg segment in Figure 8b and the airplane wing segment in Figure 8c. Moreover, Figure 8a demonstrates the model’s ability to accurately segment small and occluded cows, further highlighting its effectiveness in handling such challenging scenarios. In contrast to yolov8-seg [15], our model achieves higher segmentation accuracy and overall segmentation quality. For instance, in Figure 8a, the original yolov8-seg fails to accurately segment an overlapping cow in the distant background, while in Figure 8c, the segmentation of the airplane wing in yolov8-seg contains redundant regions. The segmentation results from various methods collectively demonstrate that the model proposed in this paper excels in enhancing the segmentation of large targets, small targets, and edge regions.

**Figure 9:**
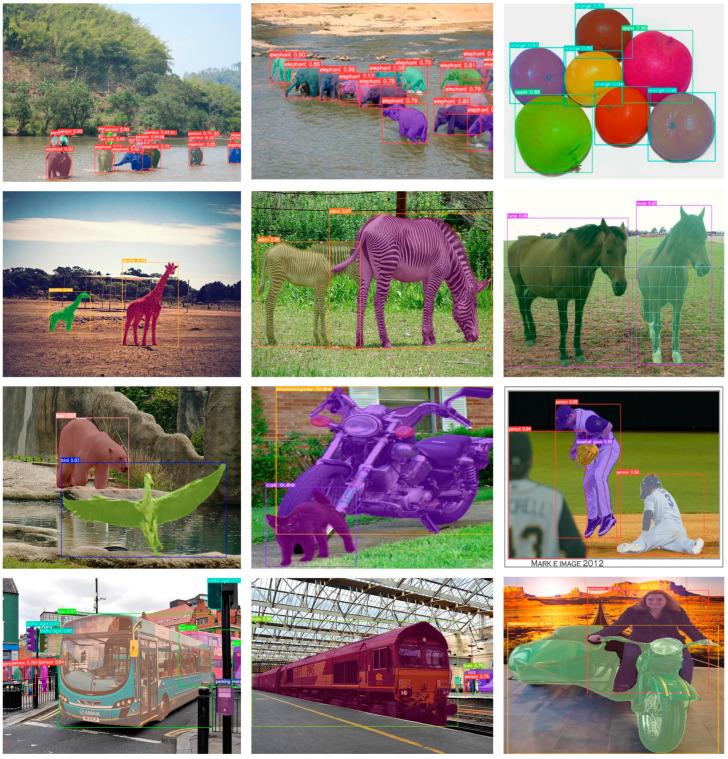
More visual results of ESAMask on the COCO val set.

To further demonstrate the segmentation effectiveness of ESAMask across a broader range of object categories, Figure 9 presents a diverse and comprehensive collection of visual examples. These examples encompass various scenes, object categories, and scales. Analysis of the figure reveals that our method excels in distinguishing between different instances of the same category and visually similar instances of different categories. Notably, in row 1 (columns 1 and 2), our method accurately discriminates between elephants. Similarly, in row 2, it successfully segments giraffes, zebras, and horses. Additionally, it effectively discriminates between shape-similar objects, such as apples and oranges, in row 1 (column 3). Importantly, our method achieves complete segmentation even for larger objects like buses (row 4, column 1) and trains (row 4, column 2) in street scenes. These visual examples convincingly showcase the generalization capability of our method in accurately segmenting objects across diverse categories and scales.

## 5. Limitation and Future Work

In this paper, we introduce sparse attention with semantic queries, which, compared to other fixed-rule window attentions, incorporates additional steps to compute the adjacency matrix of relevant regions. While this step does not result in a significant decrease in speed, it unavoidably introduces an increase in parameters and memory access frequency. Furthermore, the inclusion of MRFCPM allows for the simultaneous modeling of global, regional, and local information, thereby increasing the model’s complexity and computational cost. In the future, we will explore the application of lightweight methods such as pruning and quantization to the model, aiming to investigate more efficient sparse attention approaches. Additionally, inspired by the work of [44], we plan to study effective segmentation methods for images in challenging conditions such as rainy or foggy weather, to meet the demands of real-world application scenarios.

## 6. Conclusions

This paper presents a novel single-stage segmentation method, ESAMask, that aims to strike a better balance between accuracy and efficiency. To achieve this goal, we follow the principles of simplicity, lightweightness, and effectiveness and propose three novel modules: RSPA, GSInvSAM, and MRFCPM. These modules enable ESAMask to generate high-quality masks with lower computational cost. We extensively evaluate ESAMask and its components on the MS COCO dataset through quantitative experiments and visualization results. Our results show that ESAMask maintains fast and real-time advantages in high-accuracy segmentation. We believe that our method can contribute to the development of faster and more accurate instance segmentation in the future. 

## Figures and Tables

**Table 1:** ESAMask vs. some typical frameworks on the COCO val set. The ‘-’ means that the original official paper does not give a corresponding value. ‘Time’ represents the total pre-processing, inference, and post-processing time (ms) required to complete the segmentation of each image.

Methods	Backbone	Time	FPS	mAP	AP_S_	AP_M_	AP_L_
PANet [4]	R-50	212.8	4.7	36.6	16.3	38.1	53.1
Mask RCNN [3]	R-101	116.3	8.6	35.7	15.5	38.1	52.4
Point Rend [6]	R-101	100.0	10.0	38.2	19.1	40.6	55.7
RetinaMask [11]	R-101	166.7	6.0	34.7	14.3	36.7	50.5
PolarMask [12]	R-101	81.3	12.3	32.1	14.7	33.8	45.2
YOLACT [9]	R-101	30.3	33.0	29.8	10.1	32.2	50.1
YOLACT++ [10]	R-101	36.9	27.1	34.6	11.9	36.8	55.1
SparseInst [42]	R-50	25.0	40.0	37.9	15.7	39.4	56.9
E2EC [43]	DLA-34	33.2	30.1	33.8	-	-	-
SharpContour [33]	R-50	82.6	12.1	41.9	24.3	49.4	59.1
QueryInst [22]	R-101	163.9	6.1	41.7	24.2	43.9	53.9
Transfiner [21]	R-101	153.8	6.5	40.7	23.1	42.8	53.8
Mask2Former [24]	R-101	128.2	7.8	44.2	23.8	47.7	66.7
BoxeR [39]	R-101	80.0	12.5	43.8	25.0	46.5	57.9
NA [26]	NAT	40.2	24.9	44.5	-	-	-
DiNA [27]	DiNAT	40.0	25.0	45.1	-	-	-
YOLOv5-seg [16]	CSPDarknet	21.0	47.6	40.1	22.3	45.4	55.2
YOLOv8-seg [15]	CSPDarknet	20.9	47.8	42.6	23.5	47.3	57.8
Ours (ESAMask)	CSPDarknet	22.1	45.2	45.4	25.2	49.5	61.1

**Table 2:** ESAMask vs. some state-of-the-art methods on the COCO val set. The ‘-’ means that the original official paper does not give a corresponding value.

Methods	Backbone	Time	FPS	mAP	Params	GFLOPs
Mask RCNN [3]	R-101	116.3	8.6	35.7	135.0	-
Point Rend [6]	R-101	100.0	10.0	38.2	147.2	-
Mask2Former [24]	R-101	128.2	7.8	44.2	63.0	293.0
BoxeR [39]	R-101	80.0	12.5	43.8	40.1	240.0
NA [26]	NAT	40.2	24.9	44.5	85.0	737.0
DiNA [27]	DiNAT	40.0	25.0	45.1	85.0	737.0
YOLOv5-seg [16]	CSPDarknet	21.0	47.6	40.1	47.9	147.7
YOLOv8-seg [15]	CSPDarknet	20.9	47.8	42.6	43.8	220.5
Ours (ESAMask)	CSPDarknet	22.1	45.2	45.4	42.6	218.9

**Table 3:** Ablation of different components. RSPA: Related Semantic Perceived Attention; MRFCPM: Mixed Receptive Field Context Perception Module. The ‘√’ represents the addition of the corresponding module.

RSPA	GSInvSAM	MRFCPM	mAP	FPS	Time	Params	GFLOPs
			42.6	47.8	20.9	43.84	220.5
√			43.8	46.9	21.3	46.61	220.9
	√		43.3	48.5	20.6	38.09	201.2
		√	43.5	45.1	22.2	45.54	237.8
√	√		44.6	48.3	20.7	40.86	201.6
√	√	√	45.3	45.2	22.1	42.56	218.9

**Table 4:** Effect of M and k in RSPA. ‘M’ represents the number of divided windows. ‘k’ represents the number of relevant regions.

M	k	mAP	FPS	Time
7	4	43.9	45.1	22.2
8	4	43.8	46.9	21.3
8	6	43.9	44.8	22.3
10	6	43.6	45.2	22.1

**Table 5:** Effect of GSInvSAM structure composition on the COCO val set. ‘Base’ represents the original bottleneck structure of yolov8-seg. ‘r’ represents the expansion rate of the inverted bottleneck.

Bottleneck	mAP	FPS	Time	Params	GFLOPs
Base	42.6	47.8	20.9	43.84	220.5
GSConv + GSConv	42.1	49.1	20.4	35.10	191.1
GSConv + InvertConv (r = 2)	42.8	48.8	20.5	38.10	201.2
GSConv + InvertConv (r = 4)	42.9	47.9	20.9	40.53	209.4
GSConv + InvertConv + SAM	43.3	48.5	20.6	38.10	201.2

**Table 6:** Analysis of LKRAM kernel size on COCO val set.

k	mAP	FPS	Time	Params
Base	42.6	47.8	20.9	43.844
5	43.2	45.3	22.1	45.792
7	43.5	45.1	22.2	45.795
9	43.6	44.5	22.5	45.799
11	43.5	43.9	22.8	45.804

## Data Availability

The data presented in this study are openly available in MS COCO at https://doi.org/10.1007/978-3-319-10602-1_48 accessed on 4 June 2023.
